# GREACE-assisted adaptive laboratory evolution in endpoint fermentation broth enhances lysine production by *Escherichia coli*

**DOI:** 10.1186/s12934-019-1153-6

**Published:** 2019-06-11

**Authors:** Xiaowei Wang, Qinggang Li, Cunmin Sun, Zhen Cai, Xiaomei Zheng, Xuan Guo, Xiaomeng Ni, Wenjuan Zhou, Yanmei Guo, Ping Zheng, Ning Chen, Jibin Sun, Yin Li, Yanhe Ma

**Affiliations:** 1 College of Biotechnology, Tianjin University of Science and Technology, Tianjin, 300457 China; 2 Key Laboratory of Systems Microbial Biotechnology, Chinese Academy of Sciences, Tianjin, 300308 China; 3 Tianjin Institute of Industrial Biotechnology, Chinese Academy of Sciences, Tianjin, 300308 People’s Republic of China; 4 CAS Key Laboratory of Microbial Physiological and Metabolic Engineering, Institute of Microbiology, Chinese Academy of Sciences, Beijing, China

**Keywords:** Lysine production, *Escherichia coli*, Adaptive laboratory evolution, GREACE, Fermentation broth

## Abstract

**Background:**

Late-stage fermentation broth contains high concentrations of target chemicals. Additionally, it contains various cellular metabolites which have leaked from lysed cells, which would exert multifactorial stress to industrial hyperproducers and perturb both cellular metabolism and product formation. Although adaptive laboratory evolution (ALE) has been wildly used to improve stress tolerance of microbial cell factories, single-factor stress condition (i.e. target product or sodium chloride at a high concentration) is currently provided. In order to enhance bacterial stress tolerance to actual industrial production conditions, ALE in late-stage fermentation broth is desired. Genome replication engineering assisted continuous evolution (GREACE) employs mutants of the proofreading element of DNA polymerase complex (DnaQ) to facilitate mutagenesis. Application of GREACE coupled-with selection under stress conditions is expected to accelerate the ALE process.

**Results:**

In this study, GREACE was first modified by expressing a DnaQ mutant KR5-2 using an arabinose inducible promoter on a temperature-sensitive plasmid, which resulted in timed mutagenesis introduction. Using this method, tolerance of a lysine hyperproducer *E. coli* MU-1 was improved by enriching mutants in a lysine endpoint fermentation broth. Afterwards, the KR5-2 expressing plasmid was cured to stabilize acquired genotypes. By subsequent fermentation evaluation, a mutant RS3 with significantly improved lysine production capacity was selected. The final titer, yield and total amount of lysine produced by RS3 in a 5-L batch fermentation reached 155.0 ± 1.4 g/L, 0.59 ± 0.02 g lysine/g glucose, and 605.6 ± 23.5 g, with improvements of 14.8%, 9.3%, and 16.7%, respectively. Further metabolomics and genomics analyses, coupled with molecular biology studies revealed that mutations SpeB^A302V^, AtpB^S165N^ and SecY^M145V^ mainly contributed both to improved cell integrity under stress conditions and enhanced metabolic flux into lysine synthesis.

**Conclusions:**

Our present study indicates that improving a lysine hyperproducer by GREACE-assisted ALE in its stressful living environment is efficient and effective. Accordingly, this is a promising method for improving other valuable chemical hyperproducers.

**Electronic supplementary material:**

The online version of this article (10.1186/s12934-019-1153-6) contains supplementary material, which is available to authorized users.

## Background

Microbial strains are core element of industrial biotechnology. The rapid development of rational design methods and high-throughput screening assisted evolution systems have provided high efficiency strains for diverse industrial purposes [1–8]. Hyperproducers have been constructed to generate a large number of industrial chemicals including bulk amino acids, organic acids and other commodities, wherein many are present in over 100 g/L in final fermentation broths [9–13]. l-lysine, for example, is a microbial product predominantly used as food and animal feed additive, with approximately 2.4 million tons produced worldwide in 2015 [5]. Continuous efforts had been made to improve the production strains over the past several decades [1, 5, 14–18]. To date, the best reported lysine producers, *Escherichia coli* and *Corynebacterium glutamicum*, produced 136.5 g/L and 181.5 g/L lysine with different fermentation strategies, respectively [12, 17].

The environmental conditions at the late fermentation stage, such as in endpoint fermentation broth (EFB), is unfavourable for cell survival and metabolic activity. In addition to target chemicals produced at high concentrations, large amounts of supplements such as pH conditioners [9, 11, 12] and culture medium components such as (NH_4_)_2_SO_4_ for lysine production [12, 17] are usually added to the broth. In a previous lysine fed-batch fermentation, 136.5 g/L lysine was produced by an *E. coli* strain MU-1, and about 28 g/L NH_3_·H_2_O and 57 g/L (NH_4_)_2_SO_4_ were required to maintain the pH and provide ammonium for lysine biosynthesis [17]. High concentrations of chemicals result in high osmotic stress, which severely disturbs cell function and frequently leads to cell membrane damage [19–21]. In addition, a variety of extracellular molecules such as metal ions, organic acids, amino acids, sugars, nucleic acids, dipeptides and phospholipids are released into the broth due to cell lysis [22–24], which can perturb cellular metabolism. Therefore, host cells have to suffer complex stresses in late stage broths [19–26]. Increasing cell tolerance should be beneficial for improving production capacity.

Bacteria have naturally evolved multiple mechanisms to cope with environmental stress, such as increased synthesis of aquaporin to accelerate water export [27], regulated expression of potassium transporters to increase intracellular potassium concentration [28], expression of mechanosensitive channels [29], accumulation of osmoprotectants like glycine betaine, trehalose, ectoine and proline [30–32], and enhancement of cytoplasmic membrane stability [21]. However, these naturally evolved mechanisms are generally inadequate to prevent the strains from dramatically enhanced complex and combinatorial stress encountered in industrial conditions.

Adaptive laboratory evolution (ALE) has been demonstrated to be a powerful strategy to improve bacterial stress tolerance [33–35]. There have been some studies in many organisms to improve the stress responses and products improvement. For example, increased production of l-serine in *E. coli* was achieved through ALE in cultures with increasing amount of l-serine [36]. Elsewhere, hydrogen induced stress has been used to develop strains overproducing hydrogen in *Thermotoga maritima* [37–39]. Strain mutagenesis methods and environmental conditions provided for mutating cells and enriching mutants are two major factors affecting ALE efficiency and effectiveness. In a typical ALE process, spontaneous mutants which have survived after gradually increased stress are selected [36, 40]. In order to enhance ALE efficiency, different mutagenesis strategies have been applied, such as deletion of *mutS* or *mutL* to create a stress-induced mutagenesis system [41], utilization of a genetically modified proofreading element of the DNA polymerase complex (ε subunit encoded by *dnaQ* gene) to construct a “Genome Replication Engineering Assisted Continuous Evolution” (GREACE) system [42]. Using these methods, mutation occours continuously at high rates during the ALE process, which, on one hand, enlarges the mutation diversity and may increase the probability of selecting expected mutants. However, on the other hand, the remaining mutagenesis system in the selected mutants can unfavourably reduce the genotype stability and phenotype homogeneity of the final strains.

The environmental conditions provided for mutants survival can largely impact the effectiveness of ALE process. Usually, during microbial ALE for improving stress tolerance, a microorganism is cultivated under clearly defined condition such as M9 minimal medium or Luria–Bertani (LB) medium containing a stress-providing chemical. However, due to complex unknown interactions of living organisms with their environment, the evolutionary trade-offs are ubiquitous [33]. It was shown for *E. coli* that increased tolerance to ethanol comes at the cost of decreased resistance to acidic conditions [43] and strains evolved under phosphate limitation conditions showed trade-offs in NaCl and oxidative stress survival [44]. Therefore, for biotechnological purposes, to avoid the trade-offs as far as possible, a real living stress condition such as the late-stage fermentation broth should be chosen as the ALE environment. To the best of our knowledge, ALE in such a stress condition has not been reported.

In this study, we used the EFB as the starting evolution condition to carry out a modified GREACE-assisted ALE in order to enhance the tolerance and lysine production of strain MU-1 [17]. The improved GREACE method includes expressing a DnaQ mutant KR5-2 which can increase genome mutagenesis rate by 317-fold [42] with the control of an arabinose inducible promoter on a temperature-sensitive plasmid, so as to timely introduce mutations and stabilize acquired genotypes. A mutant RS3 was ultimately obtained, which produced 155.0 g/L lysine, improved by 14.8%. Further genomics and metabolomics analyses, coupled with molecular biology studies revealed that three genetic mutations of *speB* (agmatinase gene), *atpB* (gene of membrane subunit a of FoF1-type ATP synthase), and *secY* (gene of a subunit of preprotein translocase) may contribute to the improved stress tolerance and the enhanced metabolic flux into lysine. This study brings forward alternative targets to engineer lysine producer and provides an effective strategy involving ALE in EFB to improve production of other valuable hyperproducers.

## Methods

### Chemicals and enzymes

l-Lysine and arabinose were supplied by Sinopharm Chemical Reagent Co., Ltd (Tianjin, China). Propidium iodide (PI) was purchased from Sigma-Aldrich (USA). Ampicillin and kanamycin were supplied by Solarbio (Beijing, China), and 3-morpholinopropanesulfoinc acid (MOPS) was supplied by Amresco (USA). Other pure chemicals used in this study were of analytical grade or better. DNA polymerase was obtained from Transgene (Beijing, China). The ClonExpress II One Step Cloning kit was purchased from Vazyme (Nanjing, China). The Wizard Genomic DNA Purification Kit was obtained from Promega. Restriction endonucleases, T4 DNA kinase and T4 DNA ligase were purchased from New England Biolabs, Inc. (Beijing, China).

### Cultivation media

The fermentation medium consists of (per liter) 40 g glucose, 5 g KH_2_PO_4_, 1 g MgSO_4_, 10 g (NH_4_)_2_SO_4_, 0.003 g FeSO_4_, 0.003 g MnSO_4_, 50 g corn syrup, and 0.7 g KCl [17]. The EFB of *E. coli* strain MU-1 was collected by centrifugation at 12,000 rpm for 10 min and filtration with a 0.22 μm syringe filter. The initial lysine concentration of the EFB was approximately 136.5 g/L [17]. The EFB was diluted with water or concentrated by vacuum distillation. Thereafter, 5 g/L yeast extract and 10 g/L peptone were supplied to prepare EFB media with lysine concentrations of 75 g/L, 100 g/L, 125 g/L, 140 g/L and 150 g/L. Solid plates were prepared by adding 2% agar to the liquid medium. According to the resistance of cultivated strains, ampicillin and kanamycin were added at final concentrations of 100 mg/L and 25 mg/L, respectively.

### Plasmid and strain construction

The strains, plasmids and primers used in this study are listed in Table 1. Other strains were constructed based on them. The plasmid pAG in MU-1 [17] was cured after serial transfers in LB medium without kanamycin, and the resulting strain was denoted as MU-11. The *dnaQ* mutant *KR5*-*2* which is capable of inducing a 317-fold increased cell mutagenesis rate was amplified from the plasmid pQ-*dnaQ*-*KR5*-*2* [42] using primers KR-F and KR-R. The resulting PCR product was digested with *Eco*RI and *Sma*I, and ligated to the plasmid pKD46 (GenBank accession no.: MF287367, kanamycin resistance, temperature sensitive) digested with the same restriction enzymes (with *exo*, *bet* and *gam* genes deleted, keeping the arabinose inducible promoter). The generated plasmid was named as pKAR, which was transformed into MU-11 to generate MU-11 (pKAR).

**Table 1:** Bacterial strains, plasmids and primers used in this study

Strain/plasmid/primer	Description	Source
Strain		
LYS1	Derivative of *E. coli* MG1655, capable of producing lysine [17]	Lab stock
MU-1	Lysine hyperproducer obtained by high-throughput screening [17]	Lab stock
MU-11	Derivative of MU-1 by elimination of its plasmid pAG that is non-relevant with lysine production [17]	This study
Plasmid		
pKAR	Kanamycin resistance, substitution of the *exo*, *bet* and *gam* genes on plasmid pKD46 (GenBank accession no.: MF287367) with a *dnaQ* mutant *KR5*-*2* [42]	This study
pSB4K5-I52002	Kanamycin resistance, GenBank accession no.: EU496099	[45]
pSB	Kanamycin resistance, backbone pSB4K5, used as a control plasmid without expressing any additional gene	This study
pSB-*speB*	Derivative of pSB, expressing SpeB with native promoter	This study
pSB-*speB*^C905T^	Derivative of pSB, expressing SpeB^A302V^ with native promoter	This study
pSB-*atpB*	Derivative of pSB, expressing AtpB with native promoter	This study
pSB-*atpB*^G494A^	Derivative of pSB, expressing AtpB^S165N^ with native promoter	This study
pSB-sRNA^*secY*^-MicC	Derivative of pSB, expressing a small regulatory RNA to inhibit SecY synthesis	This study
Primer		
KR-F	cctgaattcgagctctaaggaggttataaaaaatgagcactgcaattacacgccag	
KR-R	tatcccgggttattatgctcgccagaggcaacttccgcctttc	
B4K5-F	tcaactagtagcggccgctgcaggag	
B4K5-R	cgacggatcctagggaattcgagtcac	
speB-F	gaattccctaggatccgtcgcgctgttaacccagttccgcgat	
speB-R	cagcggccgctactagttgacaatgtttgacgaccatcctgcatc	
atpB-F	gaattccctaggatccgtcgtgatagcaagtggattgctgttc	
atpB-R	cagcggccgctactagttgaatcatcgggatagcatccaccag	
secY-F	gaattccctaggatccgtctttacagctagctcagtcctagggactgtgctagcatctaatcccggttgtttagccattttctgttgggccattgcattg	
secY-R	cagcggccgctactagttgtataaacgcagaaaggcccaccc	

To construct the plasmid pSB and its derivatives, the pSB4K5 backbone was obtained by PCR amplification from the plasmid pSB4K5-I52002 [45] with primers B4K5-F and B4K5-R. The *speB* and *atpB* genes (or their mutants) together with their promoter regions were amplified from MU-11 (or its mutant) using the respective primers speB-F and speB-R, atpB-F and atpB-R. The pSB4K5 backbone was treated with T4 DNA kinase, and self-ligated using T4 DNA ligase to obtain pSB. The *speB* gene and its mutant, *atpB* gene and its mutant were ligated separately with pSB4K5 backbone via homologous recombination using the ClonExpress II One Step Cloning kit to obtain the corresponding plasmids pSB-*speB*, pSB-*speB*^C905T^, pSB-*atpB*, pSB-*atpB*^G494A^. To construct plasmid pSB-sRNA^*secY*^-MicC, primers secY-F and secY-R were used to amplify the MicC scaffold and T1/TE terminator from plasmid pEC-sRNA^*gfp*^-*hfq* [46]. The primer sec-F contained the sequences of the corresponding 1–24th bases of *secY* gene and a constitutive promoter J23109 (http://parts.igem.org/Part:BBa_J23100). The obtained fragment was ligated with pSB4K5 backbone via homologous recombination to generate pSB-sRNA^*secY*^-MicC. The plasmids were transformed separately into LYS1 to generate LYS1 (pSB), LYS1 (pSB-*speB*), LYS1 (pSB-*speB*^C905T^), LYS1 (pSB-*atpB*), LYS1 (pSB-*atpB*^G494A^), and LYS1 (pSB-sRNA^*secY*^-MicC).

### Adaptive laboratory evolution process

To improve the tolerance of MU-11 (pKAR) in its real living stress condition, diluted or concentrated EFB media were prepared for the GREACE-assisted ALE process. ALE was performed at 30 °C in 24-deep-well plates with 1 mL medium in each well, and 10 samples each in one well were subject to the ALE process. The process was initiated by cultivation of MU-11 (pKAR) overnight in LB medium with 10 g/L arabinose to induce expression of the DnaQ mutant KR5-2. The culture was used as a seed to inoculate EFB media with 75 g/L lysine, followed by two rounds of transfers in EFB media with 100 g/L and 125 g/L lysine, respectively. In the first round in EFB medium with 125 g/L lysine, 10 g/L arabinose was supplied again to induce expression of KR5-2. Afterwards, cells were transferred into EFB medium with 140 g/L lysine for one round and then into EFB medium with 150 g/L lysine for another round. Three additional parallel samples without supplying arabinose during the whole process were used as the control groups. During the ALE, transfer was conducted when the OD_600_ (optical density at 600 nm) increase reached 0.2–0.3 compared to the control groups. The ALE process was stopped when the control culture almost could not grow in a certain EFB medium wherein the samples can obviously grow. The inoculation ratio was 1%. For measurement of cell growth, 150 μL culture from each well was sampled into Corning 96-Well Microplate, and OD_600_ was measured using a microplate reader (SpectraMax 190 from Molecular Devices, China). After the final transfer, samples were cultivated at 42 °C in EFB medium with 75 g/L lysine and without kanamycin to cure the plasmid pKAR. Thereafter, cells in each well were spread over agar plates of EFB media with 75 g/L lysine, and cultivated at 42 °C. Three colonies from each well were selected and further evaluated.

For testing growth of MU-11 and the ALE selected mutants in EFB media, strains were sequentially transferred at a ratio of 1% in the broths with lysine concentrations of 75 g/L, 100 g/L, 125 g/L and 150 g/L. Cultivation was carried out at 37 °C in 24-deep-well plates with 1 mL culture in each well. OD_600_ was measured using the microplate reader as above.

### Fermentation tests

The fermentation medium was used for all the fermentation tests. Fermentation processes were carried out according to the methods described previously [17]. For fermentations in the 96-deep-well plate and shake flask, MOPS was supplemented at a final concentration of 0.4 mol/L to buffer the pH change. Lysine productions of MU-11 and its mutants in 96-deep-well plate with 300 μL culture in each well were tested at 16 h, while those of LYS1 derived strains were tested at 40 h. Fermentation tests of MU-11 and its mutants were also carried out in 500-mL flasks containing 20 mL fermentation medium shaking at 220 rpm. After cultivation for 16 h, 400 μL 25% NH_3_·H_2_O was supplied to each flask, and fermentation continued to 24 h. OD_600_ of the samples was detected using a spectrophotometer (UV-1800, Shimadazu, Japan). Glucose and extracellular lysine were detected using SBA-40D (Biosensing Analyzer, Shandong, China) as described previously [17]. For fermentation in 5-L jar fermenters, two rounds of precultures were conducted to activate strains and prepare seed cultures. For the main fermentation process, the initial volume was 1.75 L. Continuous maintenance of pH at 7.0 with 25–28% ammonia, glucose at 5–10 g/L with 500 g/L glucose solution and ammonia–nitrogen at 0.05–0.1 g/L with 500 g/L ammonium sulfate solution were performed as described previously [17].

### Cell membrane damage analysis

Cell membrane damage was analyzed by measuring the uptake of the fluorescent dye propidium iodide (PI). Cells were collected by centrifugation at 7000 rpm for 2 min. Pellets were resuspended in PBS (NaCl 8 g/L, KCl 0.2 g/L, Na_2_HPO_4_·12H_2_O 3.58 g/L, KH_2_PO_4_ 0.24 g/L, pH 7.2) to an OD_600_ of about 0.5. The suspensions were added with PI (prepared as 200 μg/mL stock solution in PBS) to the final concentration of 2 μg/mL, and incubated in the dark for 10 min at room temperature. Cells were subsequently washed once with PBS. The uptake of PI was analyzed using a MoFlo XDP flow cytometer (Beckman Coulter Inc., USA) according to a previous method [25] with minor modifications. The excitation wavelength and the emission wavelength were 488 nm and 620 nm, respectively. Data for at least 50,000 cells per single sample were collected. Percentage of positive cells for PI analysis was calculated using the statistics module of the Summit 5.2 software.

### Genome sequencing

The processes for sample preparation and genome sequencing were carried out according to a previously reported method [47]. Genomic DNA was purified using the Wizard Genomic DNA Purification Kit. The average insert size of the library was 500 bp, and the average read length was 150 bp. Sequencing was performed using an Illumina Miseq 2000 system (Tianjin Institute of Industrial Biotechnology, Chinese Academy of Sciences, Tianjin, China). The raw sequence reads were sub-sampled and trimmed by removing low quality bases. The resulted reads were aligned onto the reference genome of MU-11 (unpublished data). The alignment result was visualized for assembly. SNP and Indel variants were analyzed, and relevant gene annotation was performed.

### Intracellular metabolomics analysis

The intracellular metabolomics were analyzed as reported previously [48]. Cellular metabolism was quenched using fivefold volume of − 20 °C 40% methanol. Intracellular metabolites were extracted using ethanol/water (3:1, v/v) at 100 °C combined with acidic acetonitrile/water (1:1, v/v, with 0.1% formic acid) at − 20 °C. The LC–MS/MS analysis and data processing were all carried out accordingly.

## Results and discussion

### Construction of a GREACE-assisted ALE method for improving MU-11 tolerance in the EFB media

In a recent study, we obtained a high-level lysine producing *E. coli* strain MU-1. The EFB contained 136.5 g/L lysine, and about 28 g/L NH_3_·H_2_O and 57 g/L (NH_4_)_2_SO_4_ were supplied, leading to high stress [17]. For many fermentation processes, the product generation rates obviously decrease in the late stages, especially for products with high concentrations [11, 12, 17]. One major reason for this reduction in product formation might be the cells encounter challenging stress in the late-stage fermentation environment. It is reasonable to assume that cells bearing enhanced stress tolerance might gain a survival advantage and maintain better metabolic activity to generate more products.

**Fig. 1:**
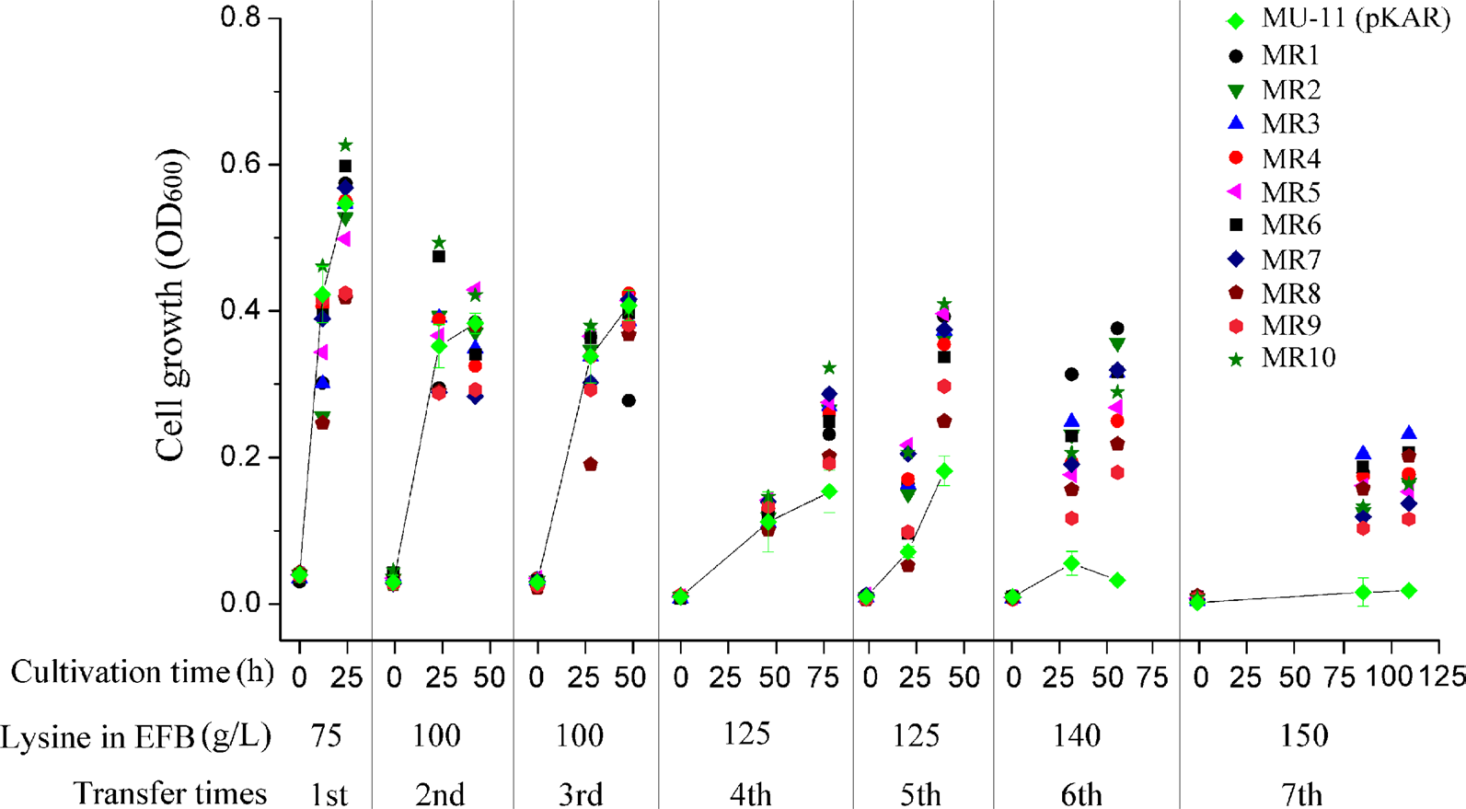
Growth of MU-11 (pKAR) and ten ALE samples in EFB media. The respective seeds for the ten samples were prepared by cultivating MU-11 (pKAR) overnight in LB medium with 10 g/L arabinose. Cells were then serially transferred in EFB media with lysine concentrations from low to high. At the fourth transfer, 10 g/L arabinose was added into the sample cultures again. For the cultivation of MU-11 (pKAR) as a control, arabinose was not used during the whole process. The inoculation ratio was 1%. The growth curves of the control groups were linked to highlight the growth differences between controls and mutants. Data for the growth of MU-11 (pKAR) are the mean and standard deviation of independent triplicates

In this study, to accelerate the process of ALE, avoid excessive mutation, and timely stabilize the genotype, we reconstructed the GREACE system by expressing the DnaQ mutant KR5-2 [42] under the control of the arabinose inducible promoter on the temperature-sensitive plasmid pKD46. The constructed plasmid pKAR was transformed into MU-11 to generate MU-11 (pKAR). As shown in Fig. 1, during the transfer of control MU-11 (pKAR) in the EFB media with lysine concentration increased from 75 to 150 g/L, the cell growth level gradually decreased. In the medium with 150 g/L lysine, MU-11 (pKAR) almost could not grow. Comparatively, the final OD_600_ of all 10 samples, named as MR1 to MR10, improved by around 10- to 18-folds in EFB medium with 150 g/L lysine (Fig. 1 and Additional file 1: Fig. S1). The whole process was performed within only 7 transfers, much fewer than previously reported ALE processes [33], indicating the high efficiency of improving bacterial stress tolerance in EFB medium using the established GREACE-assisted method in our study.

### Evaluation of ALE selected mutants by fermentation and growth test

**Fig. 2:**
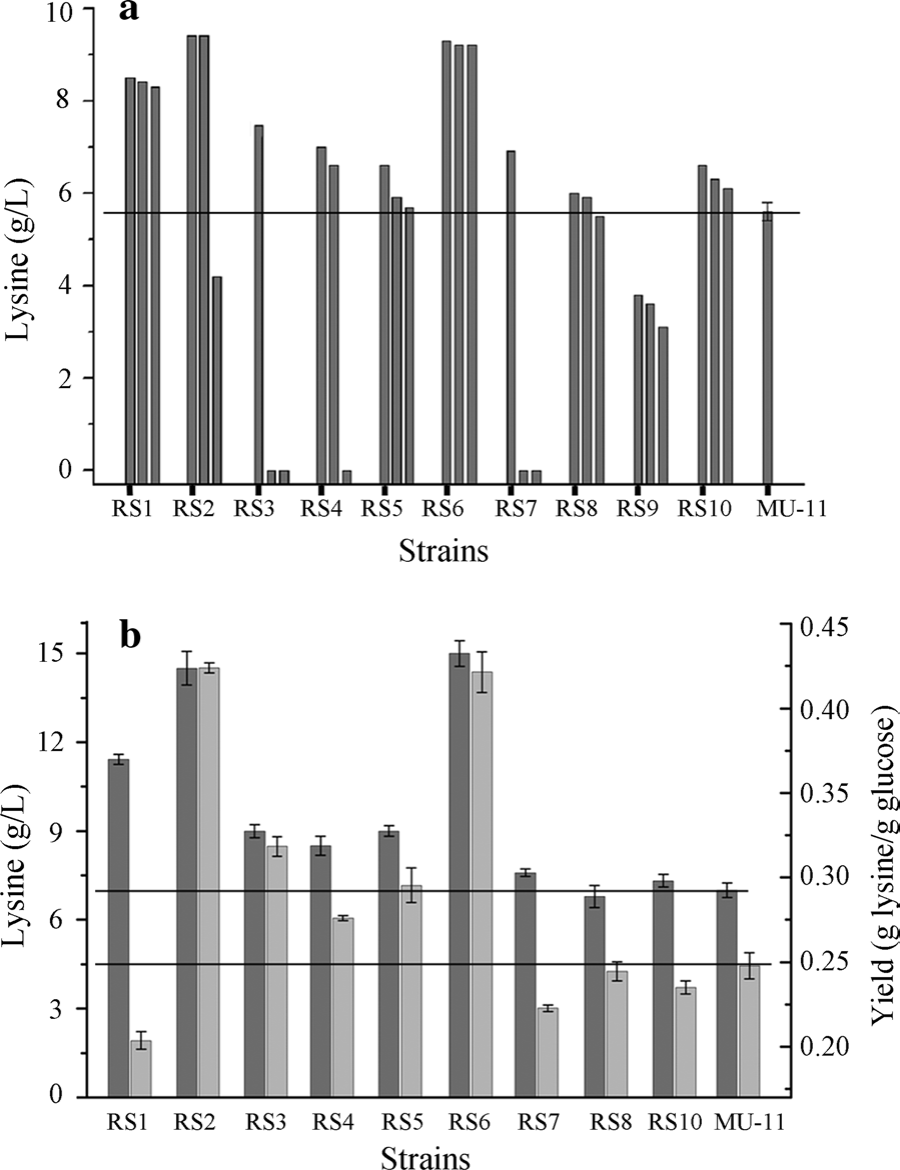
Lysine productions of ALE selected mutants in 96-deep-well plates and flasks. **a** Lysine production in 96-deep-well plates. Lysine was tested at 16 h. Three colonies from each of the 10 samples were randomly selected and tested. The three colonies in each sample were arranged in the figure according to the lysine production from high to low, their best ones were named as RS1 to RS10. Data for MU-11 are the mean and standard deviation of independent triplicates. **b** Lysine production of 9 mutants in 500-mL flasks containing 20 mL media. Dark grey bars and light grey bars represent lysine concentration and yield of lysine from glucose, respectively. pH was adjusted at 16 h using 400 μL 25% NH_3_·H_2_O, and samples were tested at 24 h. Data are the mean and standard deviation of independent triplicates

To test the lysine productivities of the evolved strains, three colonies from each of the 10 samples were randomly selected after cultivation in 42 °C to eliminate the plasmid pKAR, and the lysine productivities of the selected strains were evaluated by fermentations in 96-deep-well plates. The best ones in each of the 10 samples were named as RS1 to RS10. Nine of the 10 samples contained mutants (except for RS9) with improved lysine productions (Fig. 2a). The 9 mutants were subjected to fermentation test in flasks. By comparing the lysine titer and yield with the control strain MU-11, the mutants RS2, RS3, RS4, RS5 and RS6 showed improved lysine production (Fig. 2b).

**Fig. 3:**
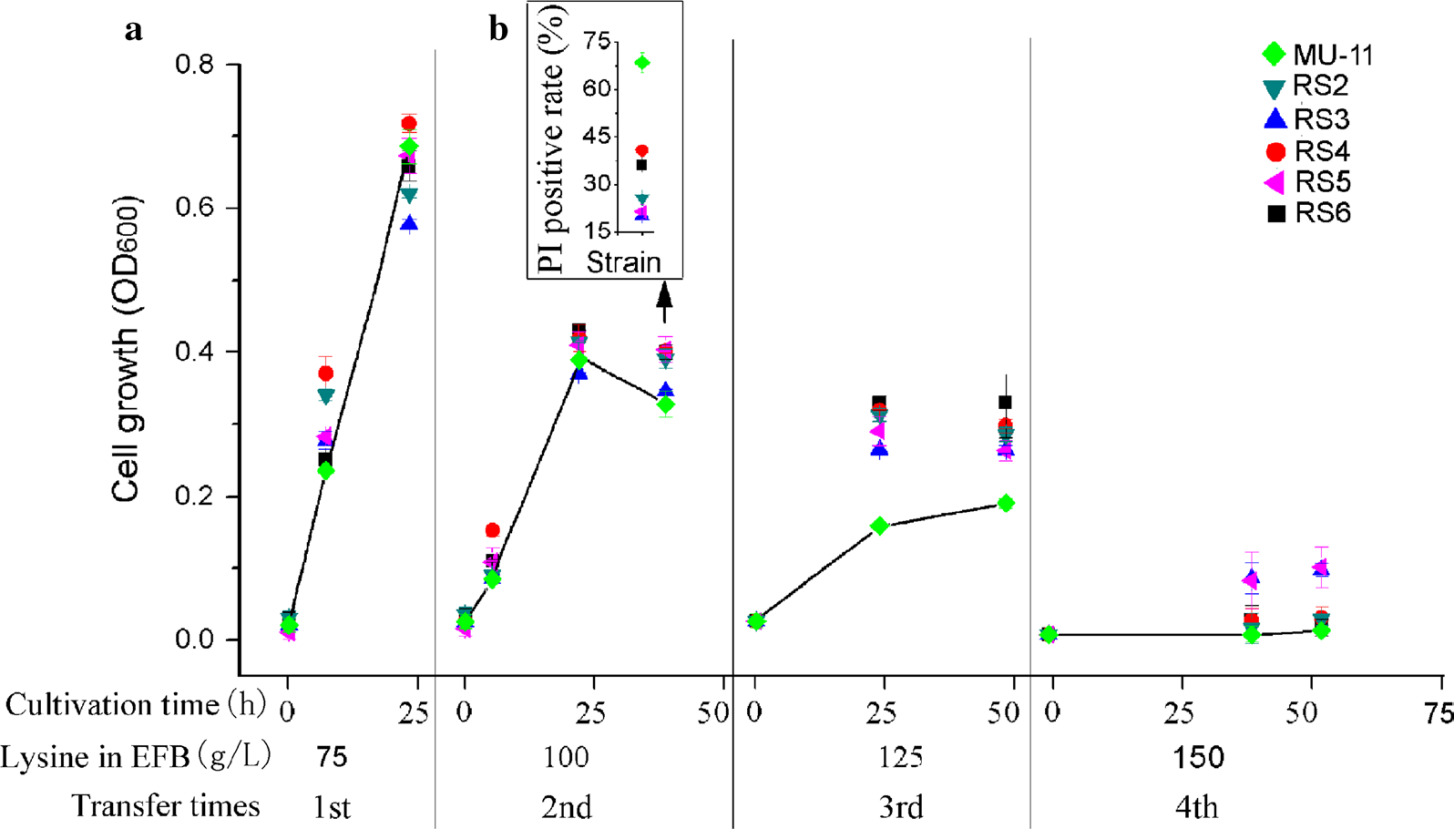
Growth tests and PI uptake analyses of MU-11 and selected mutants in EFB media. **a** Growth of MU-11 and selected mutants. Cells were serially transferred at a ratio of 1% in EFB media with lysine concentrations from low to high. **b** PI uptake of MU-11 and selected mutant cells from the 40 h-samples cultivated in EFB media containing 100 g/L lysine. The growth curves of the control groups were linked to highlight the growth differences between controls and mutants. Data are the mean and standard deviation of independent triplicates

In order to test the stress tolerance of the selected mutants with improved lysine production, cell growth of RS2, RS3, RS4, RS5, and RS6 was measured in 24-deep-well plates at 37 °C. The cells were sequentially transferred in the EFB medium with lysine concentration from low to high (75, 100, 125 and 150 g/L). As shown in Fig. 3a, The OD_600_ of MU-11 at 24 h in EFB medium with 75 g/L lysine was higher than most of the tested mutants, indicating that the initial strain with high lysine producing capacity can also resist low stress and the mutants have no advantage under the minor stress conditions. In EFB media with 100 g/L lysine, even though the cell densities of MU-11 and the mutants differed slightly at 40 h, the PI uptake analysis, which has been frequently used to measure cell membrane integrity [20], revealed that MU-11 was the most damaged strain (Fig. 3b). When the lysine concentration in the EFB medium further increased to 125 g/L, the OD_600_ increase of all tested mutants were higher than that of MU-11. These data indicate that all the tested mutants showed improved tolerance in the EFB medium, suggesting that ALE under the cell living condition such as EFB medium could be an effective strategy. When even higher stress such as EFB medium with 150 g/L lysine was applied, all cells exhibited dramatically decreased growth. However, mutants RS3 and RS5 obviously grow better than other mutants and the MU-11 control (Fig. 3a).

### Fed-batch fermentation tests and metabolomics analyses of the selected and control strains

**Fig. 4:**
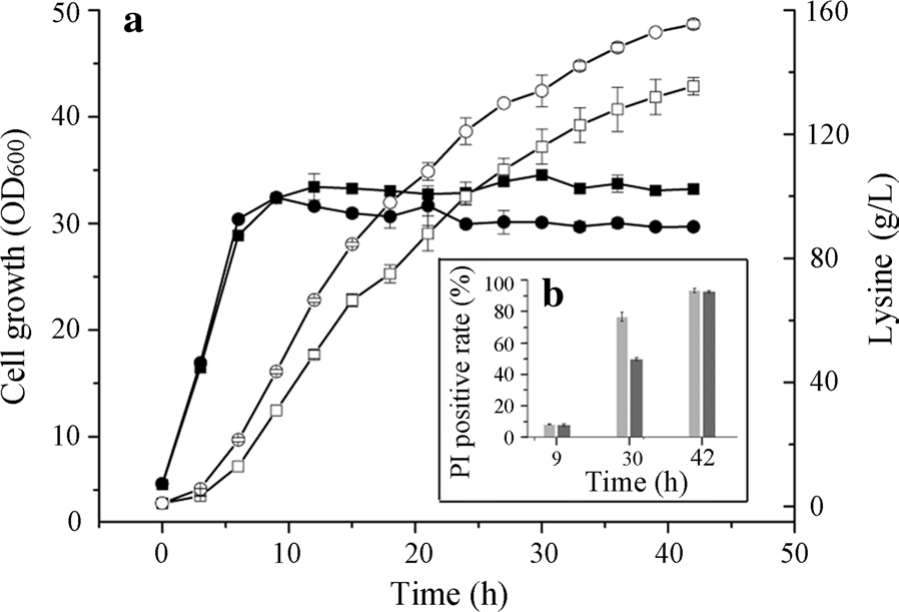
Fermentation tests and PI uptake analyses of MU-11 and RS3 in 5 L jar fermenter. **a** Lysine productions and cell growth of MU-11 and RS3. Open circles and open squares represent the lysine productions of RS3 and MU-11, respectively. Filled circles and filled squares represent the growth of RS3 and MU-11, respectively. **b** PI uptake of MU-11 and RS3 cells from the samples at different fermentation time. Light grey bars and grey bars represent the PI positive rates of MU-11 and RS3, respectively. Data are the mean and standard deviation of independent triplicates

After evaluation of fermentation performance and stress tolerance of selected mutants in deep-well plates and shake flasks, the best mutant RS3 was further examined in a 5 L jar fermenter with a mimetic industrial fed-batch fermentation process. As shown in Fig. 4a and Table 2, lysine production of RS3 was highly improved. The final titer, yield and total lysine amount in one batch reached 155.0 ± 1.4 g/L, 0.59 ± 0.02 g lysine/g glucose, and 605.6 ± 23.5 g, accounts for improvement of 14.8%, 9.3%, and 16.7%, respectively.

**Table 2:** Lysine productions of MU-11 and RS3 in 5 L jar fermenters

Strain	Lysine concentration (g/L)	Yield (g lysine/g glucose)	Total lysine in one batch (g)
MU-11	135.0 ± 2.8	0.54 ± 0.01	519.1 ± 27.9
RS3	155.0 ± 1.4	0.59 ± 0.02	605.6 ± 23.5

The PI uptake analysis was carried out to test the membrane integrity of RS3 and MU-11 during fermentation (Fig. 4b). At 9 h, a time point representing the early fermentation stage with less stress, the cell membrane damage degrees were slight for both strains. At 30 h, a time point representing mid to late fermentation stage with more than 120 g/L lysine produced, the damage degrees of both strains increased, but MU-11 was obviously worse than RS3. The result implied that under a proper stress condition, RS3 was relieved from serious cell damage. At 42 h, the end of fermentation, an extremely stressful condition, the cell membranes of more than 90% of both strains were damaged, and the degree of RS3 was a little slighter than MU-11, which might also be the reason for better growth of RS3 after a long period of cultivation in the EFB media with 150 g/L lysine (Fig. 3).

The growth of the mutant RS3 in the 5 L fermenter delayed unexpectedly after 10 h in comparison to that of the control MU-11. This presumably was not caused by stress, since both strains did not face serious stress as shown by limited membrane damage at 9 h depicted in Fig. 4b. Another mutant RS5 even suddenly stopped growth when OD_600_ reached about 19 at 6 h in a 5 L fermenter (data not shown). Similar phenomena about the growth of mutants selected by ALE in one condition showing impediment in another condition has also been reported [33]. Therefore, to evaluate the potential of the obtained mutants for industrial purpose, it is better to test their performance in jar fermenters since more variants such as glucose and ammonia–nitrogen levels, and fermentation condition control were introduced. Nevertheless, the production capacity of RS3 was apparently enhanced. Comprehensive optimization of the fermentation medium in future work would further improve the production performance of RS3 as well as RS5.

**Fig. 5:**
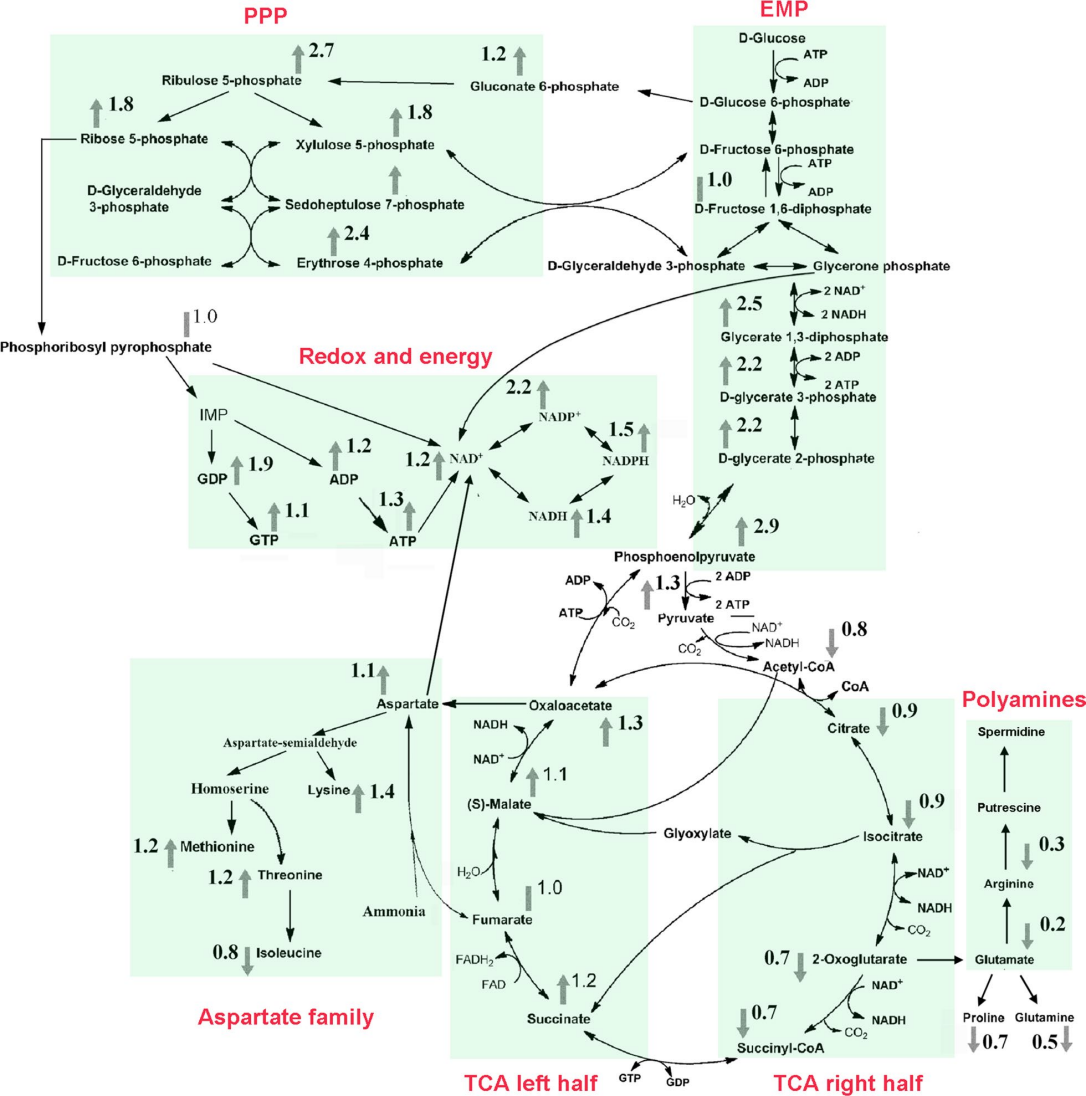
Intracellular metabolic profiling of intermediates involved in the selected metabolic pathways in RS3. Metabolic modules are marked with color. The modules include The Embden–Meyerhof–Parnas Pathway (EMP), the pentose phosphate pathway (PPP), the tricarboxylic acid cycle (TCA left half and TCA right half), the aspartate family amino acids synthetic pathways (Aspartate family), the redox equivalents and GTP/ATP synthetic pathways (Redox and energy), and the polyamines synthetic pathway (Polyamines). Samples of 9 h fed-batch fermentation were applied for the metabolomics analysis. The arrows indicate the increase (arrow pointing upwards) or decrease (arrow pointing downwards) of metabolites in RS3 compared with MU-11, while the bars without arrow mean almost no change. The values were determined by dividing the LC–MS/MS peak areas of metabolites in RS3 by the corresponding peak areas of metabolites in MU-11. Data are the mean of independent triplicates

To understand the metabolic mechanism for strain improvement, metabolomics analyses of RS3 and MU-11 cultures in the 5-L jar fermenters were carried out. In order to avoid the influence of cell damage to the analytical results, the 9 h fermentation cultures, wherein the damage degree was slight and almost the same for MU-11 and RS3 (Fig. 4b), were sampled and quenched immediately. Intracellular metabolites were extracted and then analyzed using LC–MS/MS. The LC–MS/MS data were normalized by cell density, and the concentrations of corresponding metabolites in MU-11 and RS3 were compared. As shown (Fig. 5 and Additional file 1: Table S1), compared with MU-11, the EMP, PPP, TCA left half, aspartate family amino acid synthesis, and redox equivalent and energy currency synthesis pathways of RS3 were all enhanced. The down regulation of the TCA right half pathway might be due to the cellular regulation to limit carbon flux into TCA cycle under enhanced carbon metabolism and energy generation condition [49]. The enhanced metabolic flux into lysine synthesis and increased redox equivalents especially NADPH synthesis are beneficial for lysine production [1, 14]. The improved ATP and NADPH supplies may provide more energy for cells to tolerate stressful conditions [50, 51].

### The impact of genomic variations on stress tolerance and lysine production of RS3

Whole genome sequencing was performed on RS3 to discover genomic variations, and 3,655,204 high quality reads with an average read length of 150 bp were obtained. The sequence was compared with the genomic sequence of the parent strain MU-11. Three mutations occurred on genes including *speB*, *atpB* and *secY*, leading to corresponding amino acid sequence changes (Table 3). In addition, two transposases and three hypothetical proteins were mutated, each with a base change leading to an amino acid substitution (data not shown).

**Table 3:** Genomic variants of RS3 compared with MU-11

Concerned gene	NCBI gene locus	Mutant site	Amino acid variation	Protein description
*speB*	b2937	C905T	A302 V	Arginase family enzyme
*atpB*	b3738	G494A	S165 N	FoF1-type ATP synthase, membrane subunit a
*secY*	b3300	A433G	M145 V	Preprotein translocase subunit

**Fig. 6:**
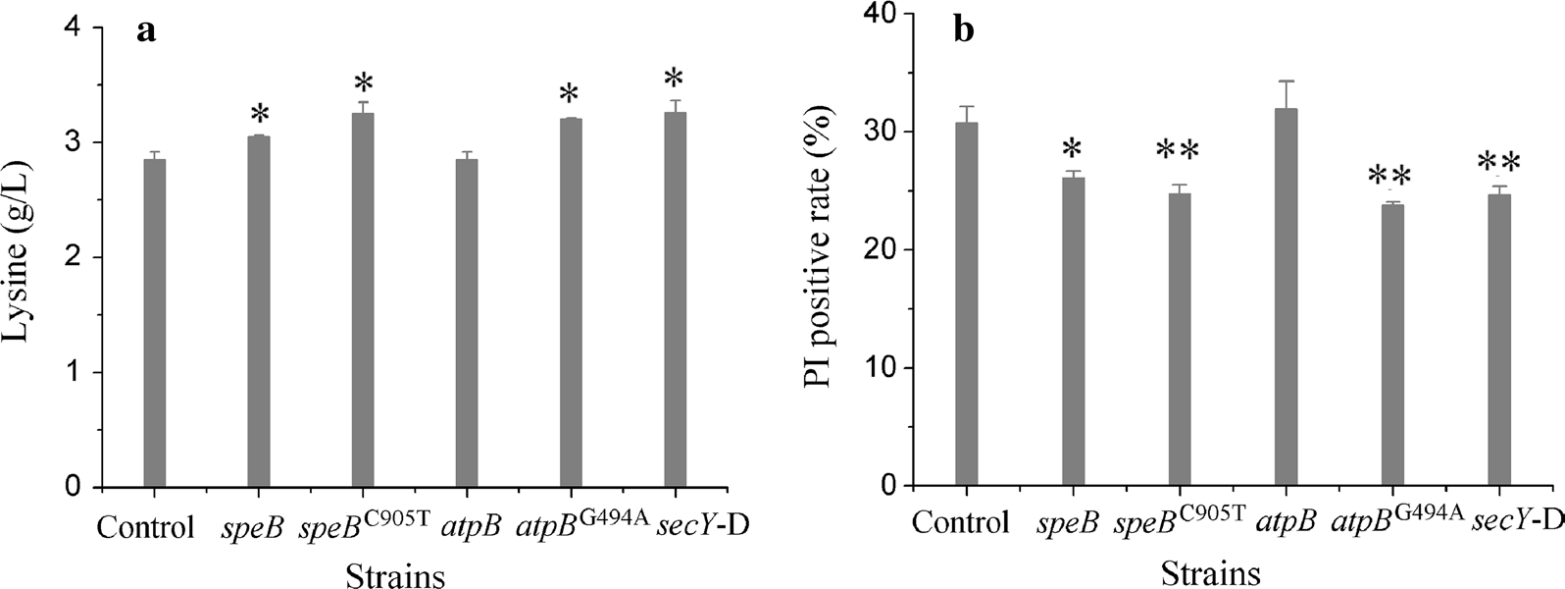
Lysine productions and PI uptake analyses of LYS1 derived strains. **a** Lysine productions. **b** PI uptakes. Control represents LYS1 (pSB). *speB* represents LYS1 (pSB-*speB*). *speB*^C905T^ represents LYS1 (pSB-*speB*^C905T^). *atpB* represents LYS1 (pSB-*atpB*). *atpB*^G494A^ represents LYS1 (pSB-*atpB*^G494A^). secY-D represents LYS1 (pSB-sRNA^*secY*^-MicC), which can transcribe a small regulatory RNA (sRNA) to inhibit SecY synthesis. Cells were cultivated for 40 h in 96-deep-well plate with fermentation medium. Data are the mean and standard deviation of independent triplicates. The p value was calculated by Student’s two-tailed t-test. * represents a p value of < 0.05, and ** represents a p value of < 0.01, comparing with the data of control strain

To evaluate the effects of A302V mutation in SpeB (corresponding to nucleotide change of C905T) (Table 3) on cellular stress tolerance and lysine production, the wild-type and mutant SpeB were over expressed respectively in strains LYS1 (pSB-*speB*) and LYS1 (pSB-*speB*^C905T^), which were subjected to fermentation test in a 96-deep-well plate incubated for 40 h. As shown in Fig. 6a, b, compared with the control strain LYS1 (pSB), LYS1 (pSB-*speB*) showed improved lysine production and cell membrane integrity. Since SpeB catalyzes the conversion of arginine to putrescine and spermidine, improved metabolic flux to polyamines biosynthesis is expected from SpeB overexpression. A previous study has demonstrated that the catabolism of these polyamines is a metabolic response to stress in bacteria such as *E. coli* [52]. Therefore, enhancing polyamine biosynthesis may be beneficial for lysine production and stress tolerance. Interestingly, overexpression of the SpeB mutant further increased lysine production and cell integrality, indicating improved performance of the SpeB mutant.

Numerous repair systems for stress induced cell damage are ATP-dependent. Improving ATP production is an important mechanism for strain tolerance [50, 51]. The enzyme AtpB is a subunit of the complex FoF1-type ATP synthase [53]. S165 of AtpB locates nearby an important site R169 whose mutation R169A can lead to the complete inactivation of the ATP synthase [54]. The effect of AtpB^S165N^ (corresponding to nucleotide change of G494A) (Table 3) on lysine production and cell damage was examined. The control strain LYS1 (pSB) and the recombinant strain LYS1 (pSB-*atpB*) overexpressing the wild type AtpB showed similar lysine production and cell membrane integrity, suggesting that overexpression of a single component of the mature ATP synthase complex might not affect the ATP synthase complex activity. However, the lysine production of LYS1 (pSB-*atpB*^G494A^), and cell integrity as well, improved (Fig. 6), indicating that the AtpB^S165N^ could affect the activity of ATP synthase and should be beneficial for RS3. This is consistent with the increased level of ATP in RS3 compared to MU-11 revealed by metabolomics analysis (Fig. 5 and Additional file 1: Table S1).

SecY forms a channel of the important membrane protein secretory system complex SecY/E/G and SecA [55, 56]. SecY alteration that impaired membrane protein folding can up-regulate the Cpx/sigma(E) stress response pathways [57], which is important for acid stress and cell wall stability in *E. coli* [58]. To test the impact of decreased SecY expression on lysine production and cell membrane damage, strain LYS1 (pSB-sRNA^*secY*^-MicC) transcribing a small regulatory RNA (sRNA) to inhibit SecY synthesis was constructed. The sRNA contains a 24 bp sequence complementary to the 1–24th bases of *secY* gene, and contains a MicC scaffold for recruiting the Hfq protein [59]. Compared with the control strain LYS1 (pSB), LYS1 (pSB-sRNA^*secY*^-MicC) showed improved lysine production and decreased cell membrane damage (Fig. 6), which is consistent with the previous report. In addition, considering the location of the M145 site in the P2 region of SecY, which might be involved at the interface with SecG [60], the M145 V mutation of SecY is more likely prone to weaken the function of the protein complex SecY/E/G and SecA.

According to the results, it could be deduced that the SpeB^A302V^, AtpB^S165N^ and SecY^M145V^ mutants are likely to be the main contributors to the improvement of RS3 (Fig. 4a). However, it was difficult to deduce the mutants induced causality of the stress tolerance (Figs. 4b, 6b) and the overall metabolic enhancement of RS3 (Fig. 5).

Taken together, ALE is effective to improve stress tolerance of microbial cell factories but a time-consuming process that commonly requires tens of transfers and takes weeks up to months [33]. Strategies to increase mutagenesis rates are helpful for accelerating the ALE process [41, 42]. In this study, GREACE that facilitates mutagenesis coupled-with selection under stress conditions was modified and applied to accelerate the ALE process. Growth of all the 10 paralleled samples improved under stress conditions after only seven rounds of transfers (Fig. 1), indicating the high efficiency of the modified GREACE-based ALE method. Another improvement to traditional ALE process is the usage of the late-stage fermentation broth to mimic the actual stress conditions that industrial producers faces. By using this strategy, mutants with significant increase in lysine production and stress tolerance were obtained. The ALE strategy established here should also be helpful for improving hyperproducers of various valuable chemicals. Genome sequencing of the best mutant RS3 provided valuable information for understanding the mechanism of improved lysine production and stress tolerance. Mutations in genes involved in polyamine biosynthesis, energy supply, and protein secretory were proven to be beneficial, which highlighted the importance of these three cellular processes in enhancing bioproduction.

## Conclusions

By reconstructing the GREACE system, we developed a mutagenesis controllable GREACE-assisted ALE process in EFB. The process was applied to improve the stress tolerance of the lysine hyperproducer *E. coli* MU-1 in lysine EFB medium. Tolerance enhanced mutants were obtained within only 7 transfers in EFB media, indicating the high efficiency of the established process. Because environmental stress is harmful to high production of chemicals by bacteria, we expect that this method established in the present study would be extended to improve other chemical hyperproducers.

## Additional file

**Additional file 1: Fig. S1.** Growth comparison of the 10 ALE samples with the control samples. **Table S1.** Partial intracellular metabolites of RS3 from the 9-h fed-batch fermentation sample.

## Data Availability

All data generated or analyzed during this study are included in this published article and the Additional file 1. The authors are willing to provide any additional data and materials related to this research that may be requested for research purposes.

## Abbreviations

ALE: adaptive laboratory evolution
GREACE: genome replication engineering assisted continuous evolution
EFB: endpoint fermentation broth
PI: propidium iodide
DnaQ: ε subunit of the DNA polymerase
SpeB: agmatinase
AtpB: membrane subunit a of the FoF1-type ATP synthase
SecY: one subunit of preprotein translocase
EMP: The Embden–Meyerhof–Parnas Pathway
PPP: the pentose phosphate pathway
TCA: the tricarboxylic acid cycle
