# Supplementary material for: GREACE-assisted adaptive laboratory evolution in endpoint fermentation broth enhances lysine production by Escherichia coli
Source: Microb Cell Fact. 2019 Jun 11;18:106. doi: 10.1186/s12934-019-1153-6 (PMC6560909; doi:10.1186/s12934-019-1153-6)
Supplement: Supplementary file 1 — Additional file 1: Fig. S1. Growth comparison of the 10 ALE samples with the control samples. Table S1. Partial intracellular metabolites of RS3 from the 9-h fed-batch fermentation sample. [file 12934_2019_1153_MOESM1_ESM.docx]

**Additional file 1.**

**GREACE-assisted adaptive laboratory evolution in endpoint fermentation broth enhances lysine production by *Escherichia coli***

Xiaowei Wang^1,2,3†^, Qinggang Li^2,3†^, Cunmin Sun^2,3^, Zhen Cai^4^, Xiaomei Zheng^2,3^, Xuan Guo^2,3^, Xiaomeng Ni^2,3^, Wenjuan Zhou^2,3^, Yanmei Guo^3^, Ping Zheng^2,3*^, Ning Chen^1^, Jibin Sun^2,3*^, Yin Li^4^, Yanhe Ma^3^

Present address: ^1^ College of Biotechnology, Tianjin University of Science and Technology, Tianjin 300457, China; ^2^ Key Laboratory of Systems Microbial Biotechnology, Chinese Academy of Sciences, Tianjin 300308, China; ^3^ Tianjin Institute of Industrial Biotechnology, Chinese Academy of Sciences, Tianjin 300308, China; ^4^ CAS Key Laboratory of Microbial Physiological and Metabolic Engineering, Institute of Microbiology, Chinese Academy of Sciences.

^*^ To whom correspondence should be addressed: Ping Zheng ([zheng_p@tib.cas.cn](mailto:zheng_p@tib.cas.cn)), or Jibin Sun (sun_jb@tib.cas.cn). Address: Tianjin Institute of Industrial Biotechnology, Chinese Academy of Sciences, Tianjin 300308, People’s Republic of China. Fax/Phone: +86-02284861943.

^†^Xiaowei Wang and Qinggang Li dedicated equally to this work


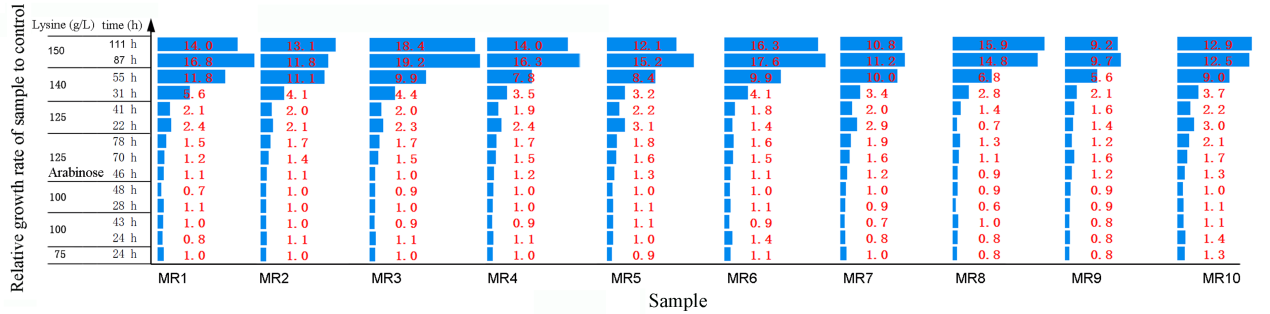


**Fig. S1** Growth comparison of the 10 ALE samples with the control samples. The relative growth of samples to controls were calculated by dividing the OD_600_ of MU-11 (pKAR) grown in EFB media supplied with arabinose in the seed culture and the first 125 g/L-lysine culture by OD_600_ of MU-11 (pKAR) grown in EFB media without supplying arabinose during the whole process. Cells were serially transferred at a ratio of 1% in EFB media with lysine concentrations from low to high.

**Table S1** Partial intracellular metabolites of RS3 from the 9-h fed-batch fermentation sample

| **Metabolite** | **Relative**  **amount^a^** | **Mean Error (ppm)** | **Mean Peak area** |
| --- | --- | --- | --- |
| D-Fructose 1,6-bisphosphate | 1.0 ± 0.5 | 0.5 | 2228.0 |
| Glycerate 1,3-diphosphate | 2.5 ± 0.9 | 14.2 | 3549.5 |
| D-Glycerate 3-phosphate | 2.2 ± 0.2 | 0.9 | 275475.7 |
| D-Glycerate 2-phosphate | 2.2 ± 0.2 | 0.9 | 275475.7 |
| Phosphoenolpyruvate | 2.9 ± 0.0 | -0.6 | 42427.4 |
| Pyruvate | 1.3 ± 0.4 | 0.1 | 3403.0 |
| Oxaloacetate | 1.3 ± 0.2 | 3.9 | 316.0 |
| Acetyl-CoA | 0.8 ± 0.1 | 1.0 | 18483.1 |
| Citrate | 0.9 ± 0.3 | -0.4 | 55801.2 |
| Isocitrate | 0.9 ± 0.3 | -0.4 | 55801.2 |
| 2-Oxoglutarate | 0.7 ± 0.1 | -0.6 | 5228.0 |
| Succinyl-CoA | 0.7 ± 0.0 | 5.3 | 690.4 |
| Succinate | 1.2 ± 0.1 | -0.1 | 388784.9 |
| Fumarate | 1.0 ± 0.1 | -0.4 | 105275.8 |
| (S)-Malate | 1.1 ± 0.1 | 0.8 | 429116.4 |
| NAD+ | 1.2 ± 0.0 | -3.5 | 10778.1 |
| NADP+ | 2.2 ± 0.1 | -3.2 | 6371.1 |
| NADH | 1.4 ± 0.3 | -9.3 | 2587.3 |
| NADPH | 1.5 ± 0.2 | 1.0 | 4616.3 |
| ATP | 1.3 ± 0.7 | -0.8 | 3365.7 |
| ADP | 1.2 ± 0.2 | -0.7 | 21194.8 |
| GTP | 1.1 ± 1.9 | 1.0 | 763.0 |
| GDP | 1.9 ± 0.5 | -0.8 | 4400.7 |
| Gluconate 6-phosphate | 1.2 ± 0.3 | 8.5 | 3080.2 |
| Ribulose 5-phosphate | 2.7 ± 0.2 | -0.9 | 81706.9 |
| Xylulose 5-phosphate | 1.8 ±0.2 | -0.9 | 81706.9 |
| Ribose 5-phosphate | 1.8 ± 0.2 | -0.9 | 81706.9 |
| Sedoheptulose 7-phosphate | 1.1 ± 0.4 | 0.2 | 3817.0 |
| Erythrose 4-phosphate | 2.4 ± 0.3 | 41.6 | 10200.9 |
| Arginine | 0.3 ± 0.3 | -1.0 | 16645.9 |
| Aspartate | 1.1 ± 0.3 | 0.4 | 35078.5 |
| Glutamine | 0.5 ± 0.1 | -73.5 | 4326.3 |
| Glutamate | 0.2 ± 0.0 | -0.5 | 21549.0 |
| Isoleucine | 0.8 ± 0.1 | -1.0 | 5966.0 |
| Lysine | 1.4 ± 0.2 | 0.6 | 819645.0 |
| Methionine | 1.2 ± 0.2 | 0.4 | 2349.8 |
| Proline | 0.7 ± 0.4 | -0.6 | 4680.0 |
| Threonine | 1.2 ± 0.7 | 66.99 | 11363.2 |

**^a^**The relative amount was calculated by dividing the peak area of each metabolite in RS3 by that in MU-11.
